# Low-value chronic prescription of acid reducing medication among Dutch general practitioners: impact of a patient education intervention

**DOI:** 10.1186/s12875-024-02351-2

**Published:** 2024-04-04

**Authors:** Joris L. J. M. Müskens, Simone A. van Dulmen, Karin Hek, Gert P. Westert, Rudolf B. Kool

**Affiliations:** 1 IQ Health Science Department, Radboud University Medical Center, Research Institute for Medical Innovation, Nijmegen, The Netherlands; 2 Nivel, Netherlands Institute for Health Services Research, Utrecht, The Netherlands

**Keywords:** Low-value care, General practice, Patient decision aid, Dyspepsia, Acid reducing medication

## Abstract

**Background:**

Dyspepsia is a commonly encountered clinical condition in Dutch general practice, which is often treated through the prescription of acid-reducing medication (ARM). However, recent studies indicate that the majority of chronic ARM users lack an indication for their use and that their long-term use is associated with adverse outcomes. We developed a patient-focussed educational intervention aiming to reduce low-value (chronic) use of ARM.

**Methods:**

We conducted a randomized controlled study, and evaluated its effect on the low-value chronic prescription of ARM using data from a subset (*n* = 26) of practices from the Nivel Primary Care Database. The intervention involved distributing an educational waiting room posters and flyers informing both patients and general practitioners (GPs) regarding the appropriate indications for prescription of an ARM for dyspepsia, which also referred to an online decision aid. The interventions’ effect was evaluated through calculation of the odds ratio of a patient receiving a low-value chronic ARM prescription over the second half of 2021 and 2022 (i.e. pre-intervention vs. post-intervention).

**Results:**

In both the control and intervention groups, the proportion of patients receiving chronic low-value ARM prescriptions slightly increased. In the control group, it decreased from 50.3% in 2021 to 49.7% in 2022, and in the intervention group it increased from 51.3% in 2021 to 53.1% in 2022. Subsequent statistical analysis revealed no significant difference in low-value chronic prescriptions between the control and intervention groups (Odds ratio: 1.11 [0.84–1.47], *p* > 0.05).

**Conclusion:**

Our educational intervention did not result in a change in the low-value chronic prescription of ARM; approximately half of the patients of the intervention and control still received low-value chronic ARM prescriptions. The absence of effect might be explained by selection bias of participating practices, awareness on the topic of chronic AMR prescriptions and the relative low proportion of low-value chronic ARM prescribing in the intervention as well as the control group compared to an assessment conducted two years prior.

**Trial registration:**

10/31/2023 NCT06108817.

**Supplementary Information:**

The online version contains supplementary material available at 10.1186/s12875-024-02351-2.

## Introduction

Dyspepsia is one of the most commonly encountered clinical conditions in general practice, with a pooled prevalence ranging between 15 and 21% of the global population [1–3]. Dyspepsia is generally defined as a symptom complex characterised by a predominant pain or discomfort in the upper abdominal region, such as epigastric discomfort or pain, heartburn or regurgitation [1]. In the Netherlands alone, approximately 800,000 patients reporting symptoms of dyspepsia annually [4]. International assessments of the prevalence of dyspepsia reveal significant variation between countries, with rates ranging from less than 1% to as high as 57% [1, 5, 6]. As dyspepsia mostly is not caused by an identifiable disease or organic abnormalities, it is generally perceived as a harmless condition, in absence of alarm symptoms such as bleeding, anaemia, unintended weight loss, or dysphagia [7–11]. 

Numerous studies have demonstrated an association between the development of dyspepsia and various lifestyle factors including diet, smoking, alcohol consumption, excessive body mass, and mental state [9–12]. Dutch guidelines for general practitioners (GPs) therefore recommend GPs to provide lifestyle advice prior to treatment with acid reducing medication (ARM), such as antacids or H2-receptor antagonist and proton-pump inhibitors (PPIs) [13, 14]. However, a recent review indicated that around a quarter of the adult population worldwide uses ARM [15]. Additionally, ARM was the most frequently prescribed drug category in Dutch general practice in 2020, with over 2.2 million users [16–19]. Although short-term ARM prescriptions are an effective way to control acid-related disease, the chronic prescription of ARM is only indicated in specific situations. According to the guidelines for Dutch GPs, chronic prescriptions of ARMs should only be considered in patients with Barrett’s oesophagus, Zollinger-Ellison syndrome, or in patients at high risk of gastrointestinal bleeding [14]. However, a recent study showed that around 88% of patients with a chronic ARM prescription in Dutch general practice lacked an appropriate indication, so called low-value prescription [20]. Although PPIs used to be considered effective and safe, there is growing concern regarding their long-term use as it is associated with numerous adverse effects such as vitamin deficiencies, development of multidrug resistance, decreased bone density, and enteric infections [21–24]. Moreover, the use of ARM can cover potential lifestyle risks. It therefore is necessary to reduce the (chronic) prescription of ARMs among Dutch general practitioners.

Previous studies have demonstrated the effectiveness of patient decision aids in reducing low-value treatment. Patient decision aids help patients comprehend the potential benefits and risks associated with their treatment options, empowering them to actively engage in healthcare decisions and make choices that align with their values [25, 26]. However, the effectiveness of the introduction of patient decision aids varies. Furthermore, in the context of chronic ARM provision, the existing evidence of their effectiveness is limited. Only one study by Krol et al., showed that the provision of patient information can effectively reduce low-value chronic ARM use through provision of an educational flyer to chronic ARM users [27]. However, the educational materials used were limited to discussing the newly updated GP guidelines on dyspepsia management and did not provide information regarding potential underlying causes, associated risks and benefits of stopping ARM use, or appropriate indications. In this study, we therefore investigated the impact of an patient focused educational intervention containing these elements on the chronic prescription of ARM.

## Methods

### Study design, phases and setting

We conducted a randomized controlled interventional study and evaluated this using data derived from a subset of practices participating in the Nivel Primary Care Database (Nivel-PCD). The Nivel-PCD contains care data routinely collected from the electronic medical records from 529 GP practices throughout the Netherlands, representing approximately 2 million registered patients [28]. Furthermore, the database contains longitudinal information regarding patient characteristics such as age, sex, GP consultations, diagnoses, and drug prescriptions. Socioeconomic status (SES) scores (on the level of Dutch postal codes) were obtained from the Central Statistical Office (CBS) [29]. Patients were assigned to one of five categories (lowest, below average, average, above average, highest) based on quintiles. Age categories were defined based on the available GP guidelines [13, 14] Diagnoses are recorded using the International Classification of Primary Care version 1 (ICPC-1). Prescriptions are recorded using the Anatomical Therapeutic Chemical classification system (ATC). This study was approved by the relevant governance bodies of the Nivel-PCD (nr. NZR00322.017) and by the Research Ethics Committee of the Radboud University Medical Centre (dossier number 2022–13,579).

### Intervention and recruitment

The intervention consisted of the distribution of a poster for the waiting room and flyers to be given to patients aiming to inform both patients and GPs with respect to the correct indications for treatment of dyspepsia (Table 1 contains an elaborate description of the intervention materials). After signing up, practices assigned to the intervention group received a package containing 60 flyers and one waiting room poster to use during consultations. The flyer and poster provide a short description of the correct indications for treatment of dyspepsia. Additionally, both the flyers and posters contained a QR-code linking to a decision aid explaining the correct indications and causes of dyspepsia. The intervention materials are added as Supplementary file 1.

**Table 1:** Intervention materials

**Web-based educational decision-aid for patients**
The educational web-based decision-aid was developed in collaboration with *thuisarts.nl (homedoctor.nl)*, a Dutch website created by the Dutch GP association. The interactive tool provides patients with information about dyspepsia and its pathogenesis and explains treatment options as well as conservative management. The aim is to reassure patients, to give patients insight in their complaints and to learn them what they can do themselves to reduce complaints.
**Flyers and posters for patients**
Flyers and posters were available to raise awareness about appropriate care in dyspepsia and inform patients about the available decision-aid. A QR-code led patients directly to the online tool.

### Sample size calculation

Based on a z-test sample size calculation using the proportion of patients that received an inappropriate chronic ARM prescription observed in an earlier assessment in the Netherlands (88% of chronic ARMs users do not have an indication), an alpha of 0.05, power of 0.80 and an expected reduction of 10%, a minimum number of 28 GP practices (with a mean of 328 patients that are inappropriately using a chronic ARM) were required to achieve significance [20]. 

### Randomisation

The participating general practitioners were recruited in a blinded manner from the Nivel-PCD. Meaning that the GPs were approached by the Nivel-PCD without receiving information regarding the purpose of the study. After having consented to participation, GPs were randomly assigned to either the intervention or control group. When a GP was assigned to the intervention group, the entire practice was seen as being exposed. GPs assigned to the intervention group received the poster and flyers, to be shared with the patients suffering from dyspepsia. GPs assigned to the control group received nothing. However, it is important to note that the access to the decision aid was not limited to the GPs of the intervention group and their patients, it was freely accessible to anyone through the website Thuisarts.nl [30]. 

### Assessment of the low-value chronic prescription of acid reducing medication

Our assessment of the amount of ARM users was conducted using a patient-indication lens, as described by Chalmers et al. [31] Implying that all patients that were chronic ARM users were included in our denominator and all patients without indication for chronic use in our numerator. Individuals were considered chronic ARM users when they had received acid reducing medication for at least 180 days in the previous year. We defined a patient’s chronic prescription as being of low-value when for at least 75% of all prescription days there was no clear indication for chronic ARM prescription present [20]. Supplementary file 2 contains an overview of the way we operationalised our assessment of low-value chronic ARM prescription. This part of the analysis was performed using STATA 16 [32]. 

### Statistical analysis of the difference in prescribing over the two periods

To assess the differences in ARM prescriptions we compared the incidence rate of (inappropriate) chronic ARM prescriptions in the same 6 months before and after the intervention (i.e. last 6 months of 2021 and last 6 months of 2022). Our primary outcome therefore would be the odds ratio (OR) of patients receiving a low-value chronic ARM prescription between the pre- and post-intervention periods. For this purpose, we built a multilevel binomial model, with an interaction term between both the indicator of cohort (i.e. 2021 vs. 2022) and an indicator indicating whether a patient was part of a practice belonging to the intervention or control group. We aimed to include random effects for both the patient and practice level when possible. However, we ended up using models only including a practice level because of the limited number of observations on the level of the patient. Generalised variance inflation factors (GVIF) were calculated to test for collinearity among the included variables before multilevel analysis was conducted (Supplementary file 3). Patient age, socioeconomic status (SES) and sex were included as case-mix variables in the models, since previous research has shown they could affect the amount of care a patient requires, receives or has access to [33–35]. Patients for which either the age or socioeconomic status was unknown were excluded from the multilevel analysis, but were included in the table showing the general description of both cohorts (as presented in Table 3). Following our analysis of the baseline characteristics of the included population, we were forced to exclude patients above the age of 80 from this analysis while no cases of low-value care provision were present, which would result in too little variation on the practice level. We therefore chose to exclude patients aged 80 and above from our analysis, prioritising the recognition of clustering at the practice level over the inclusion of this age group in our model. The pre-intervention period (2021) was taken as reference period. A *P*-value smaller or equal to 0.05 was considered statistically significant for all analyses, based on two-sided testing. Data analysis and visualisation was performed using R (version 4.4.2) [36]. 

## Results

A total of 24 practices responded to our call for participation within the recruitment period. These 24 practices were randomly assigned to either the intervention or control group, resulting in 13 practices in the intervention group and 11 practices in the control group. To even out the number of practices in each of the groups, the two practices were randomly selected from the Nivel-PCD to be added to the control group, resulting in a total of 26 participating practices. These additional practices were selected based on the similarities in size and degree of urbanisation compared to the other practices included in our analysis. Tables 2 and 3 provide a general overview of the characteristics, and recorded number of (low-value) episodes in both the intervention and control group. The initial outcomes indicate a slight increase in chronic low-value ARM prescriptions for both the control and intervention groups. In the control group, the proportion of patients with a low-value chronic ARM decreased from 50.3% in 2021 to 49.7% in 2022, and in the intervention group, it increased from 51.3% in 2021 to 53.1% in 2022. Most patients were prescribed PPI’s as subsequent analysis of the types of ARMs used over both periods revealed that the majority of patients used a PPI. In the 2021 and 2022 cohort, 99.7% and 99.3% of the patients received an PPI (ATC-codes starting with A02BC), while 2.1% and 2.4% of patients were prescribed another ARM. Furthermore, 35% of the ARM users included in the 2021 cohort were also present in the 2022 cohort. Conversely, 37% of patients included in our 2022 cohort were also present in the 2021 cohort. Our results also show that the number of prescription increases with age, however the proportion of inappropriate prescribing decreases. This can be explained by the notion that with increasing age, the number of indications for appropriate chronic ARM use also increases. Analysis of the VIF factors before performance of the multilevel analysis revealed that little or no collinearity exists among the variables included in our analysis (Supplementary Table 3).

**Table 2:** General overview of patient characteristics over both the 2021 and 2022 cohorts

Variables	Control group		Intervention group	
	2021	2022	2021	2022
Median no. of patients per practice [25–75 percentile]	3,148 [2,743 – 4,007]	3,038 [2,609 – 4,194]	2,801 [2,492–4,079]	2,771 [2,433–4,055]
Average age [± SD]	40.0 [± 23.3]	40.2 [± 23.3]	42.2 [± 21.1]	42.3 [± 23.1]
Average socioeconomic status [± SD]	0.087 [± 0.23]	0.089 [± 0.23]	0.047 [± 0.21]	0.046 [± 0,21]

**Table 3:** Cohort characteristics of both the control and interventions groups

Variables	Control group				Intervention group			
	2021		2022		2021		2022	
	No. of patients with a chronic prescription	No. of patients with a low-value chronic prescription	No. of patients with a chronic prescription	No. of patients with a low-value chronic prescription	No. of patients with a chronic prescription	No. of patients with a low-value chronic prescription	No. of patients with a chronic prescription	No. of patients with a low-value chronic prescription
***No. of patients with a chronic ARMs prescription***	1,982	996 (50.3%)	1,894	942 (49.7%)	1,733	889 (51.3%)	1,694	899 (53.1%)
*% female*	54.1%	54.6%	53.3%	54.8%	62.2%	54.7%	54.8%	56.1%
**No. of patients per age category**								
* 0–49*	229	225	229	227	155	152	165	163
* 50–59*	299	291	258	255	275	271	270	266
* 60–69*	456	328	438	302	452	333	435	326
* 70–79*	555	152	560	158	487	133	492	144
* 80 +*	443	0	409	0	364	0	332	0
**No. of patients per SES category**								
* Lowest*	359	178	315	164	587	335	545	324
* Below average*	269	139	545	241	319	158	298	160
* Average*	678	327	392	183	146	93	131	77
* Above average*	339	158	294	142	601	266	636	298
* Highest*	337	194	348	212	80	37	84	40

Subsequent multilevel regression analysis revealed that albeit the proportions showing to have slightly increased in both the control and intervention group. no significant difference in low-value chronic ARM prescription between the two groups was observed. The odds of receiving a chronic low-value ARM prescription showed to not- significantly differ when comparing the control to the intervention group over the examined periods (Odds ratio: 1.11 [95% CI: 0.84–1.47], *p* > 0.05). Table 4 contains an overview of the study outcomes after removal of the patients aged 80+, and Table 5 contains the odds ratio resulting from the subsequent statistical analysis.

**Table 4:** Overview of study outcomes. The number of chronic ARM users for each of the periods, including the proportion of these that receive a chronic low-value ARM prescription

	2021-Total	2021-Low-value	% Low-value	2022-Total	2022-Low-value	% Low-value
Control	1,539	996	64.7%	1,485	942	63.4%
Intervention	1,369	889	64.9%	1,362	899	66.0%

**Table 5:** Overview of the outcome of our analysis of the impact of our intervention on the odds of receiving an low-value chronic ARM prescription over the compared periods. The table contains both the proportions of chronic ARM users that received a low-value chronic ARM and the subsequently calculated odds ratio

	Proportion low-value 2021 (%)	Proportion low-value 2022 (%)	Odds ratio of receiving a low-value ARM between control/intervention over 2021/2022 + [95% CI]
**Control**	64,7%	63,4%	1.11 [0.84–1.47]
**Intervention**	64,9%	66,0%	

## Discussion

Our study shows that over the last half year of 2021 and 2022 in both the intervention and control group approximately half of the patients received low-value chronic ARM prescription. This indicates that ARM was still regularly prescribed over the investigated periods. Furthermore, no significant difference in the number of patients receiving a low-value chronic ARM prescription was observed between the control and intervention group (Odds ratio: 1.11 [0.84–1.47], *p* > 0.05). Additional analysis revealed that in both the 2021 and in the 2022 cohort, the majority of patients used a PPI (ATC-codes starting with A02BC, prescribed to 99.7% and 99.3% of the patients respectively while only 2.1% and 2.4 of patients in either the 2021 and 2022 cohort were prescribed another ARM). This suggests that it is highly unlikely that the lack of an effect following our intervention cannot be ascribed due to a large proportion of patients stepping down from an PPI to antacids.

### Comparison with other research

It seems that the intervention in itself did not alter the inappropriate prescription of ARM among the included GPs. This finding is not unique, however there is quite some variation in the effectiveness of similar interventions addressing low-value ARM prescribing using a patient educational tool exists.

In a study of Boster et al., the treating primary care physicians directly discussed the appropriate indications for ARM use with their patients. Using this method, they successfully reduced the patients’ ARM dosage or completely stopped ARM usage in 44% of the identified ARM users within a military hospital over a 6-month period [37]. Apart from this one study, most studies regarding the reduction of ARM use rely on providing patients the tools needed for appropriate self-management of their dyspepsia. These tools included the provision of intensive support by a specialist nurse, the formulation of an action plan and an explanation of the appropriate indications as well as the benefits of decreasing or discontinuing ARM usage. However, the outcomes of these studies vary. For example, both the study by Murie et al. and the study by Coyle et al. managed to stop or reduce PPI use (by 83% and 35%, respectively) by providing patients the tools for self-management of their ARM use, such as formulating an action plan and providing information regarding appropriate ARM use [38, 39]. Conversely, the study by Dibly et al. provided similar support to ARM users, but their study did not show to change ARM use among the included patients [40]. This observation is consistent with a previous study by Batuwitage et al., which demonstrated that providing education to patients about the appropriate indications for ARM use did not lead to a significant change in ARM utilisation [41]. However, it is worth noting that none of these studies specifically focused on chronic ARM users in their intervention evaluation. As previously mentioned, only the study by Krol et al., specifically assess the impact of their intervention on chronic ARM users, and managed to reduce chronic ARM use by 24% in the intervention group compared to 7% in the control group (24% reduction vs. 7%, respectively) [27]. The difference between our study outcome and theirs can probably be that in our study the practices assigned to the control group in our study could also had access to the intervention materials, while these were freely accessible online. This could have led to exposure of the control practices to the intervention, which was not possible in the study by Krol et al., since they only actively approached the intervention practices. This difference could explain why we did not observe a difference in low-value chronic ARM prescribing between the control and intervention groups.

### Analysis absence of effect

Our intervention did not lead to a significant reduction in low-value chronic ARM prescriptions between the intervention and control group. The present study does show a much lower percentage of low-value chronic ARM users compared to a previous assessment. Our earlier study, which examined chronic ARM use from 2016 to 2019, found that approximately 88% of chronic ARM users in the Netherlands lacked an indication. In the current study, this baseline was 66% [20]. Several possible reasons could explain the lower baseline for the included practices. First, since our previous assessment, a lot of (media) attention such as reports by national newspapers and an item during the eight o’clock news, has been generated on the appropriate use of ARM. Also, the publication of a report by the Dutch National Health Institute discussed the state of (appropriate) care provision for patients with dyspepsia early in 2021. This public attention might have had an effect on the prescription of ARM by GPs [4]. Second, the overarching national campaign started well before our distribution of the intervention materials among the intervention practices. Therefore, we cannot guarantee that before onset of our assessment the included practices (in both control and intervention groups) were not already affected. Third, the participating practices might already have affinity with improving the quality of care provision as they willingly joined the study unaware of the research topic or intervention. These practices might therefore already have a critical attitude towards the (chronic) prescription of ARMs, providing an explanation for the lower baseline observed in our study. Fourth, contact with the different intervention practices a few months after having distributed the materials revealed that the degree of exposure to the intervention varied amongst the intervention practices. Most GPs indicated that they were aware of the existence of the decision-aid. However, we do not know to what extent all GPs in the intervention practices have used the materials when seeing patients with dyspepsia. The fifth and final reason which could explain the absence of an effect following our intervention could be that our intervention was not sufficiently tailored to be effective. Hence, our intervention focussed on explaining the potential causes of dyspepsia and appropriate indications for ARMs use to both GPs and patients. However, as previous research indicated, the provision of low-value care is often the result of an interplay of multiple factors existing on multiple levels (e.g. the patient, healthcare provider and organizational or even medical society context) [42, 43]. Additionally, it shows that the effectiveness of deimplementation strategies and interventions depend on contextual factors, such as workplace culture or economic factors. Factors which we could not control in our intervention. Potentially, our intervention could have shown an effect if we had proactively put more emphasis on the use and implementation of the materials as well as improving knowledge of the existing guidelines. While in the current setup, our intervention heavily relies on the pro-active participation of the participating healthcare providers to improve ARM prescribing; something which has proven hard to monitor.

### Strengths & limitations

A strength of this study is that it used routinely collected administrative data containing high-quality and clinical information. This use of highly detailed data enabled us to accurately differentiation between appropriate and inappropriate prescriptions of ARM among patients. However, our study is also prone to limitations. Firstly, we were unable to reach the required number of practices to achieve significance according to our power calculation. Despite extensive efforts, we only managed to include 26 of the required 28 practices, making it challenging to draw definitive conclusions regarding the effectiveness of our intervention. Second, there were also some methodological limitations regarding our assessment of low-value chronic ARM prescription among GPs, as discussed in our previous study [20]. There is an inherent uncertainty in identifying whether a prescription is of low-value. Recommendations contain terms that do not map directly to data variables; also, diagnosis and procedure codes may not precisely identify patients for whom care is of low value. For instance, the recommendations regarding chronic ARM use lacked enough detail or required variables which are absent in the data to accurately distinguish appropriate from inappropriate prescribing. An illustrative example is the guideline stating that gastro-protection using a non-selective non-steroidal anti-inflammatory drug (NSAID) is justified if a patient is using a high dosage of a NSAID. However, information regarding the dosage of the prescribed NSAIDs was unavailable in the data used. We were also unable to identify patients suffering from chronic heartburn, as we only had access to diagnosis established within data of the years (and one year prior) included in our analysis. Patients diagnosed with heartburn outside of this period could therefore potentially be missed. More crucially, heartburn often only persists until patients take ARM (albeit via a prescription or obtained over the counter). The use of ARM often resolves the patients’ symptoms, resulting in the removal of the heartburn diagnosis from their medical records, making defining chronic heartburn challenging. Third, unfortunately we are unable to monitor the number of patients that actually accessed or used the monitor following a visit to their GP. We did contact participating practices to obtain an indication of whether or not patients used the decision-aid. Unfortunately, the participating GPs indicated that they did not have insight into whether the patients actually did use the decision aid and reported that patient never mentioned its use in any of the subsequent visits. Finally, the persistent relatively high prevalence of inappropriate chronic ARM prescriptions could be attributed to the perception of ARMs as relatively harmless. ARMs are readily available over the counter at most drugstores in the Netherlands. Thus, it is likely that our assessment still underestimates the true extent, as we could not capture all chronic ARM users in this study, particularly those using non-prescription ARMs.

## Conclusion

Our educational intervention did not result in a change in the low-value chronic prescription of ARM, suggesting that (low-value) chronic prescribing ARM remains an important issue in current medical practice. Future research therefore should focus on what is needed for practices to successfully adopt the use of a patient-centred decision aid and reduce low-value chronic prescribing ARM.

### Supplementary Information

**Supplementary Material 1.**

**Supplementary Material 2.**

**Supplementary Material 3.**

## Data Availability

The data that support the findings of this study are available from the Nivel, but restrictions apply to the availability of these data, which were used under license for the current study, and so are not publicly available. However, the data as presented in this study are available from the authors upon reasonable request and with permission of the Nivel.

## Abbreviations

ARM: Acid Reducing Medication
ATC: Anatomical Therapeutic Chemical
CBS: Central Statistical Office
CI: Confidence Interval
GP: General Practitioner
GVIF: Generalised variance inflation factors
ICPC-1: International Classification of Primary Care version 1
Nivel-PCD: Nivel-Primary Care Database
OR: Odds Ratio
PPI: Proton Pump Inhibitor
SES: Socioeconomic status
SD: Standard deviation
