# Supplementary material for: Low-value chronic prescription of acid reducing medication among Dutch general practitioners: impact of a patient education intervention
Source: BMC Prim Care. 2024 Apr 4;25:106. doi: 10.1186/s12875-024-02351-2 (PMC10996147; doi:10.1186/s12875-024-02351-2)
Supplement: Supplementary file 1 — Supplementary Material 1. [file 12875_2024_2351_MOESM1_ESM.docx]

**Supplementary file 1: overview of the campaign materials**

**
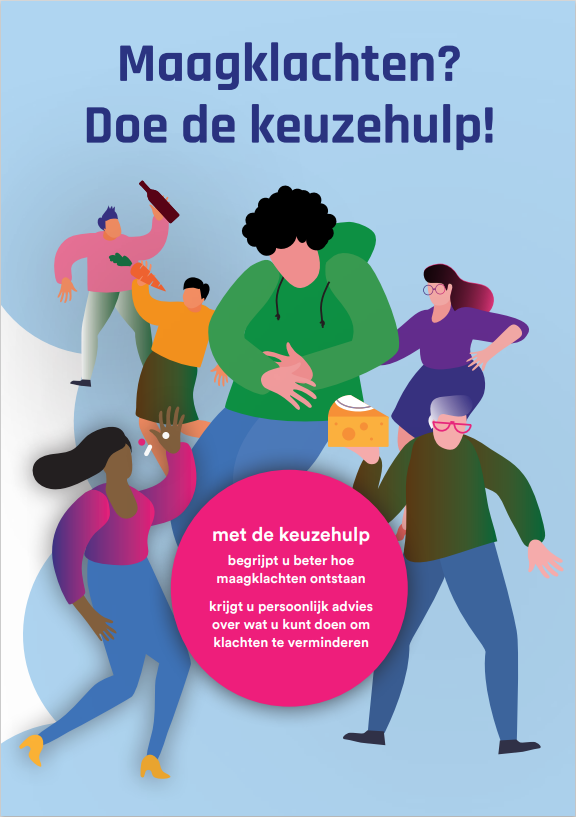
**

Figure 1: Front view of the folder used in our intervention.

**
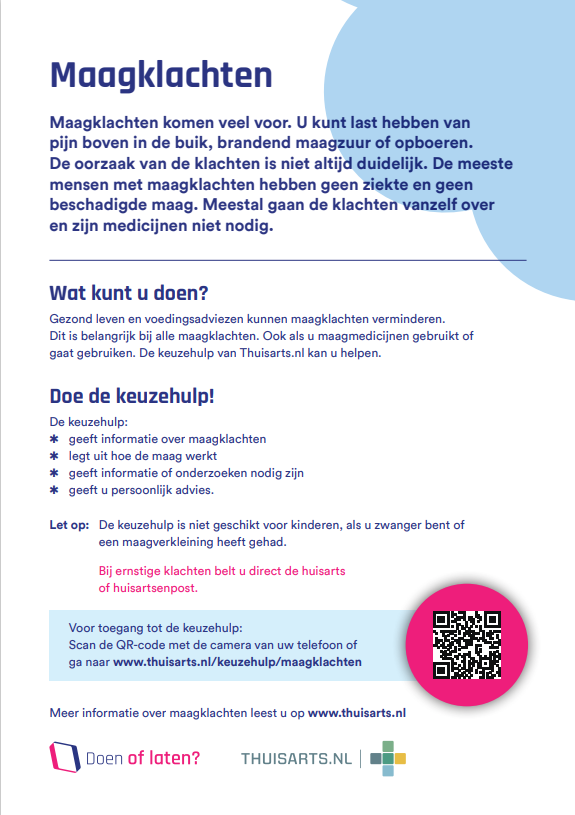
**

Figure 2: Back view of the folder used in our intervention.

**
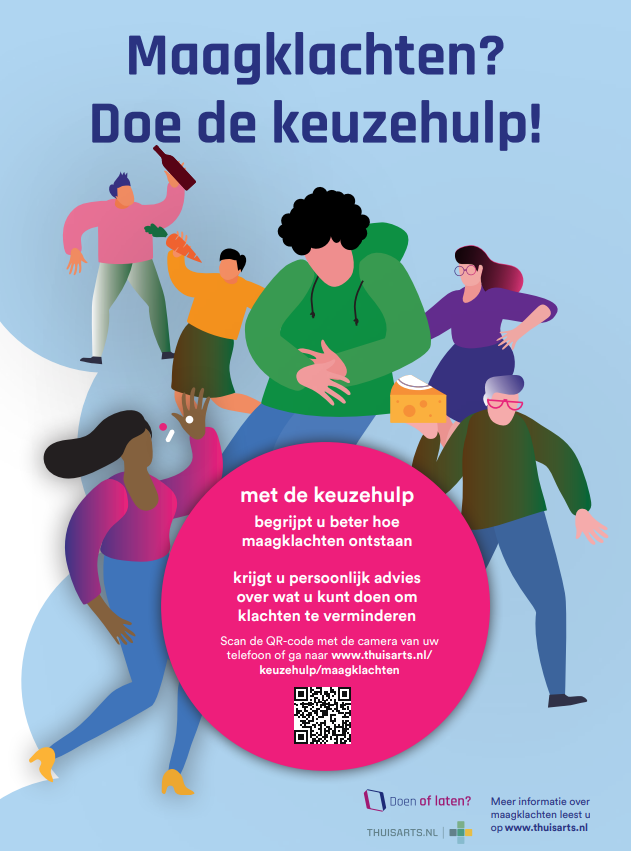
**

Figure 3: Example of the poster used in the intervention.
