# Supplementary material for: Low-value chronic prescription of acid reducing medication among Dutch general practitioners: impact of a patient education intervention
Source: BMC Prim Care. 2024 Apr 4;25:106. doi: 10.1186/s12875-024-02351-2 (PMC10996147; doi:10.1186/s12875-024-02351-2)
Supplement: Supplementary file 2 — Supplementary Material 2. [file 12875_2024_2351_MOESM2_ESM.docx]

**Supplementary file 2: detailed description the underlying recommendation and their operationalization.**

Table 1: ATC and ICPC codes used to define the study populations for each of the examined recommendations.

| **Recommendation** | **Additional information** | **ICPC codes relevant for the analysis** | **ATC codes relevant for the analysis** |
| --- | --- | --- | --- |
| 1. Do not chronically prescribe or continue acid-reducing medication (ARM), without proper indication.  - Numerator: no. of patients with a chronic prescription of ARM. - Denominator: no. of patients with no clear indication for their chronic ARM prescription. - A more elaborate description of the different conditions in which ARMs are indicated is presented below this table. | - We defined chronic acid-reducing medication users as individuals that received an acid-reducing medication for at least 180 days in the previous year. - We were unable to identify chronic ARM users suffering from chronic heartburn. - We were unable to identify patients using high-dose high dose non-selective NSAID, while no clear definition of high-dose was present in the guideline nor did we have information regarding doses of prescriptions. | D85: Duodenal ulcer D86: Peptic ulcer other D03: Heartburn L88: Rheumatoid / seropositive arthritis K77: Hearth failure T90: Diabetes non-insulin dependent | **Co-medications associated with increased risk of stomach complications:**   - - - - Coumarin derivative (B01AA)       - Direct oral anticoagulants (B01AF02, B01AE07, B01AF03, B01AF01)       - P2Y12-inhibitors (B01AC04, B01AC22, B01AC24)       - Acetylsalicylic acid derivatives: Acetylsalicylic acid (Aspirin): A01AD05, B01AC06, B01AC56, C10BX01, C10BX02, C10BX04, C10BX05, M01BA03, N02BA01, N02BA51, N02BA71.       - Systemic glucocorticoids (H02AB)       - Selective serotonin reuptake inhibitors (SSRI) (N06AB)       - Venlafaxine (N06AX16)       - Duloxetine (N06AX21)       - Trazodone (N06AX05)       - Spironolactone (C03DA01)   **Acid-reducing medication:** A02BA: H_2_-receptor antagonists A02BB: Prostaglandins  A02BC: Proton pump inhibitors M01AB: Acetic acid derivatives and related substances M01AE52: Naproxen and misoprostol |

**More elaborate description of the ARM recommendation.**

Do not chronically prescribe or continue acid-reducing medication (ARM), without proper indication. ARM prescriptions are indicated in the following cases according to the guideline:

- - Gastro-protection with an proton pump inhibitor (PPI) in case of a non-selective nonsteroidal anti-inflammatory drug (NSAID)
    1. Age of 70 years or older;
    2. Presence of an Ulcus Duodeni (D85) or Ulcus pepticum (D86) in their medical history, irrespective of their age.
    3. When two or more of the following factors are applicable (the risk of complications increases with increasing number of factors present):
       - Age between 60 and 70 years.
       - Severe disabling rheumatoid arteritis (L88), Hearth failure (K77) or diabetes (T90).
       - Use of high dose non-selective NSAID
       - Use of comedication which increase the risk of stomach complications.
  - Gastro-protection with an proton pump inhibitor (PPI) in case of a Acetylsalicylic acid derivative as platelet aggregation inhibitor and in absence of a non-selective NSAID is indicated in case of:
    1. Age 80 years or older.
    2. Age 70 or older combined with use of comedication which increases the risk of stomach complications (except Acetylsalicylic acid derivatives).
    3. Age of 60 or older combined with the presence of an Ulcus Duodeni (D85) or Ulcus pepticum (D86) in their medical history.
  - Patients suffering from chronic heartburn, which do not sufficiently benefit from alternative acid-reducing medication (or in which these have not been tried).
    1. Heartburn (D03); we could not identify patients with chronic heartburn because we only received data from the requested data period and therefore only could identify patients which received a diagnosis of chronic heartburn within the 4 years examined.

**Calculation of prescription duration**

Within Nivel-PCD only the dates on which the general practitioner prescribed the medication in question is recorded. The database does not contain the end date of a certain prescription. Therefore, in order to be able to define chronic use, we used the following method to calculate prescription duration :

When patients only have had a single prescription, we assumed these patients only received a ‘start prescription’. In the Netherlands, a start prescription has a duration of 15 days and since there is only one prescription registered, we assumed that these patients only received this single prescription and therefore only received acid-reducing medication for the duration of 15 days.

**15 days**

**Prescription date 1**

**Prescription duration of 15 days**

However, when a patient has received more than one prescription we have made the assumption that in between both dates the patient used the prescription continuously when the in between time is less or equal to 180 days. We chose to use 180 days since most prescriptions have a duration of 90 days, but medication is not always picked up after exactly 90 days. Furthermore, to assure that we also included patients that use their medication every other day instead of daily or patients which use their medication as needed we chose to double the prescription duration (e.g. 2 x 90 days) in order for those patients to be included. An additional 90 days are added to the latest prescription date of the two prescriptions, while a regular refill prescription has a duration of 90 days and the latest date of the two prescription dates us also the start date of the second prescription.

**Prescription date 1**

**Prescription duration of 188 days**

**Prescription date 2**

**90 days**

**98 days**

The same principle applies to a patient whom has 3 prescriptions, but who’s prescriptions are less than 180 days apart. The duration between prescription 1 and 2 and the duration between prescription 2 and 3 are added up, and an additional 90 days are added for the duration of the third prescription.

In case a patient has more than 180 days between consecutive prescriptions, the calculate will be performed in the following manner:

**Prescription date 2**

**90 days**

**14 days**

**45 days**

**Prescription date 1**

**Duration prescription 1: 135 days**

**Duration prescription 2: 14 days**

**Prescription date 3**

When two prescriptions are more than 180 days apart, then a new prescription will commence which in turn will be treated according to the same rules as described above.
