# Supplementary material for: Low-value chronic prescription of acid reducing medication among Dutch general practitioners: impact of a patient education intervention
Source: BMC Prim Care. 2024 Apr 4;25:106. doi: 10.1186/s12875-024-02351-2 (PMC10996147; doi:10.1186/s12875-024-02351-2)
Supplement: Supplementary file 3 — Supplementary Material 3. [file 12875_2024_2351_MOESM3_ESM.docx]

**Supplementary file 3: overview calculated generalized variance inflation factors (GVIF).**

Table 1: Generalized Variation Inflation Factors (GVIF) calculated for the model made.

|  | **GVIF** | **Degrees of freedom** | **GVIF^(1/(2*Df))** |
| --- | --- | --- | --- |
| **Studygroup (control/intervention)** | 1.672684 | 1 | 1.293323 |
| **Cohort (Cohort2021/Cohort2022)** | 1.932681 | 1 | 1.390209 |
| **Gender** | 1.010708 | 1 | 1.005340 |
| **Age category** | 1.013297 | 3 | 1.002204 |
| **Socioeconomic status category** | 1.011037 | 1 | 1.005503 |
| **Interaction Studygroup*Cohort** | 2.593739 | 1 | 1.610509 |
